# Assessment of Diagnostic Imaging Sector in Public Hospitals in Northern Jordan

**DOI:** 10.3390/healthcare10061136

**Published:** 2022-06-18

**Authors:** Ammar A. Oglat

**Affiliations:** Department of Medical Imaging, Faculty of Applied Medical Sciences, The Hashemite University, Zarqa 13133, Jordan; ammar.oglat@yahoo.com or ammara@hu.edu.jo; Tel.: +962-796-311-835

**Keywords:** advanced medical imaging, radiologic technologists, medical imaging services, CT and MRI unit

## Abstract

The most effective diagnostic methods in the medical field are diagnostic imaging techniques such as radiography, computed tomography (CT), magnetic resonance imaging (MRI), and nuclear medicine, which are used to visualize internal body to diagnose it, determine potential treatment, and evaluate and forecast care results. Therefore, the purpose of this research is to assess the diagnostic imaging sector, at three major public hospitals in the northern part of Jordan, according to regional and global requirements. The assessment approach was based on knowledge of the accessibility of diagnostic imaging equipment and its quality assurance and performance, the quantity and efficiency of radiological technologists, and the design of radiology units and medical imaging chambers in many aspects based on the use of two tools, a questionnaire and checklists, to accomplish a comprehensive evaluation. The response rate of radiological technologists was 66%. The assessment reveals a noticeable increase in the number of radiological technologists in general with high academic qualification level. Additionally, the number of diagnostic imaging equipment in Jordan revealed a large deficiency in the population–device balance, and through checklists that evaluated both CT and MRI units, it was revealed that the rate of following global requirements and occupational health and safety (OHS) standards was high. The basic supplies available in both the CT and MRI units alike were high, which indicates the high quality of healthcare provided in Jordan.

## 1. Introduction

There has been accelerated technological development in imaging techniques in many disciplines, including medicine and the healthcare system [1]. This development began with scientific breakthroughs in 1895, including the X-ray discovered by the physicist Roentgen, who was awarded a Nobel Prize for his achievement in 1901 [2,3,4,5,6,7]. Through the X-ray technique, which is named radiography, it is possible to visualize the body from the inside and diagnose several diseases and abnormalities [8]. Many technologies using ionizing radiation have appeared with the advancement of technology such as gamma rays in nuclear medicine and X-rays in mammography, computed tomography, bone densitometry, fluoroscopy, conventional radiology, and interventional radiology [6,9,10]. On the other side, there are different technologies using non-ionizing radiations such as MRI and US [11,12,13].

There are specialized chambers for conducting clinical evaluations, including medical imaging, according to the type of clinical evaluations needed and patient access to the location, as the locations and design of the chambers in the units of hospitals have been extensively studied in order to ensure the safety and comfort of the patient and the medical staff, as well as the possibility of continuous improvement and updates to keep up with the latest technology without structural obstacles [14]. Additionally, the efficiency and qualifications of radiological technologists play a major role in high-quality medical imaging processes. Hence, good results and follow-up concerning the patients’ health condition without problems are necessary in order to provide health care that complies with the global health requirements. The countries of the world, including Jordan, have witnessed a rapid development in keeping pace with medical imaging techniques and health care in general during recent decades, and this is a good indicator of public health.

Therefore, the purpose of this research is to assess the current situation in 2020 of the diagnostic imaging equipment, specifically CT and MRI, at three major public hospitals in the northern part of Jordan, where minor and major operations are carried out, in order to estimate the strengths and shortcomings of the general healthcare structure, according to regional and global requirements with regard to a country located in the Middle East region.

## 2. Material and Methods

### 2.1. Study Design and Participants

This study was revised and approved by the Institutional Review Board (IRB) at the Faculty of Applied Medical Sciences, The Hashemite University, Zarqa, 13133, Jordan (#16-11-1902402). This cross-sectional self-administration survey was carried out from September 2021 to March 2022 and distributed by sending the questionnaires to hospitals and giving them to respondents. The design of this study was used for data collection and is descriptive, empirical, and cross-sectional, with a bi-angulated configuration (quantitative and qualitative). The researchers used an arbitrated checklist to evaluate medical imaging facilities. A census study was conducted on all radiologic technologists and medical imaging departments at three main governmental hospitals in North Jordan.

The language of the questionnaire was Arabic, anonymous, and pre-structured. Moreover, it was evaluated by the experts mentioned before in the fields of radiology to ensure content validity. After that, it was pre-tested by a selected sample of radiologists to ensure visibility and face validity. Before this study began, it was tested with volunteer radiographers from the other hospitals in Jordan. An explanation of the study’s target, ensuring voluntariness and anonymity, and extended contact information of the major investigator were all on the cover page of the questionnaire.

### 2.2. Study Population and Data Collection

Three aspects were highlighted in this study: diagnostic imaging equipment, radiological technologists, and the design of radiology units and medical imaging chambers at the three main public hospitals according to regional and global requirements.

Through observing hospitals and quantifying the volume of equipment and the number of radiological technologists serving on them, the statistical results were acquired directly. Radiological technologists working in three major hospitals in northern Jordan were evaluated on both the X-ray unit and the magnetic resonance unit based on their age, gender, academic qualifications, professional certifications, general and specialized training courses, years of work and experience, and other aspects.

Medical diagnostic equipment was also evaluated based on its type, generation, model, current status, annual breakdown, and type of image that can be imaged. A checklist was applied to verify whether all requirements were achieved in accordance with global requirements such as the “Australasian Health Facility Guidelines”, the “radiological protection Institute of Ireland”, and the “National Health Services”.

### 2.3. Sample Size

Sample size was calculated by applying the Raosoft sample size calculator, which is available online. This calculation depended on the number of radiographers size in the three different hospitals. The response distribution is 50%, with a 95% level of confidence and a 5% margin of error. However, the last number of enrolled participants was 100 radiographers, to account for any lost data or non-response averages.

## 3. Result and Discussion

Table 1 represents the classification of the two types of diagnostic imaging equipment and the total number of radiological technologists working on it separately at three public hospitals in North Jordan Strip. The largest number of diagnostic imaging devices are accessible at KAUH, despite a significant deficiency of radiologic technologists operating in the MRI unit. In Yarmouk Hospital, there is only one CT scanner with a large number of radiological technologists working on it, and there is no magnetic resonance imaging at all. This is not sufficient in relation to the segment of the population that it serves and the need for possibly urgent tests that cannot be performed on a CT and require a device such as an MRI device. The highest number of radiological technologists on CT unit was at Princess Basma Hospital. This ratio is logical considering the number of CT units at Princess Basma Hospital, which provides medical and radiological services to the largest population area in North Jordan Strip.

**Figure 1:**
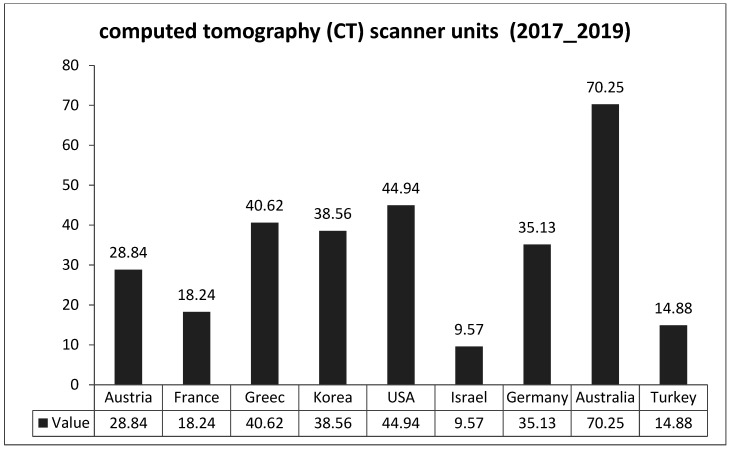
Distribution of computed tomography (CT) scanner units in the selected countries /per million population.

**Figure 2:**
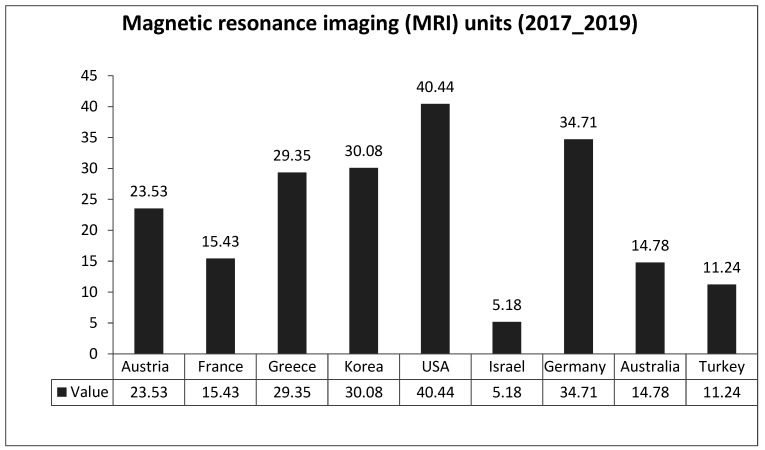
Distribution of Magnetic resonance imaging (MRI) units in the selected countries (Total/per million population).

Figure 1 and Figure 2 describe the distribution of computed tomography (CT) scanners and magnetic resonance imaging (MRI) units per million populations in selected countries. There was a significant difference between Jordan and other countries in the current readings, primarily with France, Austria, Germany, Japan, Greece, the USA, and Australia in CT units; mainly with Australia, France, Austria, Greece, Korea, Germany, and the USA in MRI units. The latest readings provide evidence of Jordan’s significant lack of medical devices in terms of both CT and MRI units.

Furthermore, 100 questionnaires were distributed to radiologists to conduct a survey and determine the most important personal and professional data that have a direct and indirect impact on the diagnostic radiology sector. These include: age, gender, academic qualifications, practical experience, training courses, and the workplace. The response rate was 66%, which is a good response; however, it is insufficient, as this response indicates a lack of awareness and real interest in scientific research in the diagnostic radiology sector at the present time.

Table 2 represents the distribution of radiological technologists’ demographics by characteristics variables in Jordan; it shows that the percentage of female radiological technologists is small, about 22%, compared to the percentage of males, which is about 78% in the diagnostic imaging sector in general. This is due to many social and health reasons related to the nature of the work, its hours, and risks in the short and long term.

These ratios are the opposite of those in the past, according to an international study conducted in 1995. It turned out that the percentage of males was about 30–40%, and the majority were females at an average rate of 60–70% [15], and the reasons for this change are as follows:

1. Unit heads tend to appoint males over females because they are not likely to take emergency leave such as pregnancy, childbirth, and postpartum leave.
2. Women rejected the arduous nature of work in the evening and night periods, as night work did not match the nature of their lives in Arab societies.
3. Women have anxiety concerning risk from exposure to high doses of ionization radiation, which may in addition impact pregnancy and potential offspring in the future.
4. Some universities inside Jordan and outside do not have a specialization in radiology technology.
5. There may be an absence of female role models in the diagnostic radiology category.
6. The significant absence of patient interaction is often elevated as a reason why women do not choose radiology as a specialty, since physicians talk to each other constantly.
7. Female high school students lack knowledge specifically of the urgent need for women specializing in the field of radiology.

The variation in the ages of radiological technologists can also be noted in Table 2, as the majority of radiological technologists (about 65%) are between the ages of 30 and 40 years, while the percentage of those who are less than 30 does not exceed 18%, which is the same for those over 40 years old. This rich diversity in the ages of radiologists leads to the units’ higher efficiency, as beginners receive guidance and knowledge from those who are older than them and more experienced than them, because the work is characterized by arduous duties and requires extensive experience.

Furthermore, Table 2 shows that most radiological technologists (around 80%) hold a bachelor’s degree as a qualification, while about 10% of them hold a diploma, and about 10% of them hold a master’s degree in equal proportion. These results contrast significantly with previous statistics that were conducted before 1996, as most radiological technologists at that time held a diploma, and a very limited number hold a bachelor’s degree. This change is due to several reasons, the most important of them being:

- Programs were established in the field of diagnostic radiology with a bachelor’s degree awarded by several Jordanian universities, along with several annual conferences that provide the most important scientific research and confirm the importance of the field in general and its effective role in the healthcare system.
- Some students obtained their university degrees from abroad and returned to work in the public hospital sectors.

Table 3 represents the amount and routine tasks of radiological technologists working on CT systems in three main public hospitals, KAUH, Yarmouk Hospital, and Princess Basma Hospital, as each radiological technologist was performing 10, 7, and 7 tasks, respectively, in the morning shift. KAUH and Princess Basma Hospital have at least two radiological technologists each, and there is one in Yarmouk Hospital in the evening and night shift.

Princess Basma Hospital performs the largest number of examinations, and this is commensurate with the number of radiological technologists present, who are divided equally between the two CT devices. This is due to the availability of two specialized advanced devices, one for cardiac and vascular exams and one for emergency. It is also clear that there is a decrease in the number of radiology technicians working in KAUH relative to the number of daily tasks, although the daily tasks are close to Princess Basma Hospital. It is worth noting that the lowest number of examinations was in Yarmouk Hospital, and this is a logical consequence of the presence of a single CT device in the hospital, as the table indicates a lack of radiological technologists on the evening and night shift.

Table 4 confirms the quantity of radiological technologists for their daily routines in the MRI units at three major public hospitals. Six radiological technologists are present in the morning shift for Princess Basma Hospital, and three radiological technologists are present in the morning shift for KAUH, but in the evening shift, there are no MRI tests at all hospitals. It is clear from the table that in Yarmouk Hospital, there are no radiological technologists working in the MRI unit, because there is no MRI device in the entire hospital, which is an urgent problem that must be solved soon by the hospital administration.

In Table 5, the statistical values of the tests that were performed are included. The statistical Pearson correlation test was applied to the relationship between the number of daily tests conducted on the CT device and the annual breakdowns of the device. The results were “correlation value” −0.198 and *p*-values of 0.687 (>0.05). These findings indicate that there is no important association between the annual breakdowns and the quantity of regular CT tests at the level of significance a −0.05.

It is logical that the more tests are performed on the CT device, the more the device malfunctions. However, through the statistical results, there was no clear effect, and the reasons are probably as follows:

- Usually, devices are selected with strong specifications and high efficiency with the lowest possible percentage of expected faults, to avoid future problems and implications.
- Some devices are fairly recent, and many tests have not been conducted on them, such as the MRI device at KAUH Hospital, which was installed at the end of 2018.
- Finally, a qualified team was present in the medical imaging unit that can deal professionally with the machines and ensure good distribution of cases throughout the working day in order to preserve them for the longest possible period without breakdowns.

In respect to the association between the assignments for tests and the number of tests in the morning shifts, the coefficient value was 0.330 and the *p*-value was 0.476 (>0.05), which suggests that there is no real connection between the assignments for exams and the number of tests at the level of significance a −0.05.

Most hospitals have had CT equipment in a functional state. Also, KAUH Hospital has lately been supplied with a CT scanner, and unrestricted cases have been authorized to be performed owing to the extensive expertise of CT analysis specialists.

### 3.1. Computed Tomography (CT) Design Checklist

Table 6 describes how CT chambers in hospitals handle global health requirements. It is evident that CT chambers have wide control console space (≥6 m^2^). The room area requirements are that the imaging room should be (≥42 m^2^) and rooms for documentation and patient recovery should be (≥8 m^2^) (AusHFG, 2016), and they have been fulfilled. Furthermore, all units have changed cubicles, and the entrance of the patient should be (≥180 × 150 cm). These have also been fulfilled. Lastly, as per global standards, medical imaging units require necessary drainage of sewage to dump the chemicals and patient waste products, and this requirement has been complied with in all units [16,17].

Table 7 demonstrates that all CT units in public hospitals have lead aprons, lead protective glasses, lead-shielded walls (thickness ≥ 3 mm and height ≥ 2 m) and doors, gonadal shielding, adequate light, radiation warning signs on the door, air conditioner systems, and good ventilation, and the persons entering the rooms are also checked by competent authorities according to global requirements [14,18,19,20].

### 3.2. Magnetic Resonance Imaging Design Checklist

Table 8 explains how MRI units handle the global health requirements at two public hospitals, KAUH and Princess Basma Hospital. It is demonstrated that MRI units in both of these public hospitals provide special forms for procedures, headphones for patients, change cubicles, and contact device “loudspeakers” between radiological technologists and patients. In addition, control consoles and imaging rooms achieved the required default dimensions of (≥10 m^2^) and (≥42 m^2^), respectively, to accommodate MRI devices and a wide locker for preserving radiofrequency coils [14].

The limitations of this study are diverse, such as the absence of revealing causality; applying snowball sampling, which weakens the potency to popularize the results; and that this study may exhibit self-chosen bias due to the method of recruiting participants that may risk both internal and external legality.

## 4. Conclusions

The response rate of the radiological technologists was not at the highest level, as it was 66%, but it was an adequate response due to the lack of radiological technologists in general. The most important results from this evaluation were a noticeable increase in the number of radiological technologists in general compared to the years before 2000 and in the ratio of males to females with the increase in the academic qualification of most workers in this sector. The amount of diagnostic imaging equipment in Jordan revealed a large deficiency in the population–device balance, and it is also reported that most of the devices/units suffer from overload of work. It also shows, based on the checklists that evaluated both CT and MRI units, a high rate of following global requirements, of following occupational health and safety (OHS) standards, and of basic supplies available in both the CT and MRI units alike, which is a good indicator of the quality of healthcare provided in Jordan.

## Figures and Tables

**Table 1:** Distribution of advanced medical imaging and the total number of RTs working on CT and MRI units at public hospitals in North Jordan Strip.

	No. of RTs/CT	No. of RTs/MRI
KAUH	8/2	5/2
Yarmouk Hospital	10/1	0
Princess Basma Hospital	12/2	6/1

**Table 2:** Distribution of RTs’ demographics by characteristics variables in Jordan.

Variables	Variables	Frequency	%
Gender	Male	14	78
	Female	4	22
Age group	<30 years	3	18
	30–40	11	65
	40–50	3	18
Qualifications	Diploma	2	10
	Bachelor’s degree	16	80
	Master’s degree	2	10
Experience	<10 years	4	22
	10–17	8	44
	17–24	4	22
	>24 years	2	11

**Table 3:** Number of daily examinations to the number of RTs in CT.

Item	KAUH	Yarmouk Hospital	Princess Basma Hospital
Number of RTs in morning shift	4	3	6
Number of exams in morning shift	40	22	45
The number of exams for each RT in morning shift	10	7	7
Number of RTs at evening and night shift	2	1	3
Number of exams at evening and night shift	10	8	20
The number of exams for each RT in evening shift	5	8	7

**Table 4:** Number of daily examinations per the number of RTs in MRI.

Item	KAUH	Yarmouk Hospital	Princess Basma Hospital
Number of RTs in morning shift	3	0	6
Number of exams in morning shift	12	0	18
The number of exams for each RT in morning shift	4	0	3
Number of RTs in evening and night shift	0	0	0
Number of exams in evening and night shift	0	0	0
The number of exams for each RT in evening shift	0	0	0

**Table 5:** The relationship between annual breakdowns in CT and number of daily examinations and between examination appointments and number of exams at morning shifts.

Machine	Test Statistic	*p*-Value *
Annual breakdowns and number of daily examinations	−0.198	0.687
Examination appointments and number of exams at morning shifts	0.330	0.476

* Correlation is significant at the 0.05 level (2-tailed).

**Table 6:** Checklist for structure of CT at public hospitals. (No. of samples = 10).

Item	KAUH		Yarmouk Hospital		Princess Basma Hospital		Total			
	Yes	No	Yes	No	Yes	No	Yes	%	No	%
Is control console area ≥ 6 m^2^?	√		√		√		10	100%	0	0%
Is room size ≥ 42 m^2^?	√		√		√		10	100%	0	0%
Is there a toilet inside the room?	√		√		√		10	100%	0	0%
Is there a recovery room for patients?	√		√		√		10	100%	0	0%
Is there a reporting room ≥8 m^2^?	√		√		√		10	100%	0	0%
Is there a change cubicle?	√		√		√		10	100%	0	0%
Is the patients’ entry ≥ 180 × 150 cm?	√		√		√		10	100%	0	0%
Is there a drainage sewage	√		√		√		10	100%	0	0%

**Table 7:** Checklist for occupational health safety (OHS) of CT at public hospitals. (No. of samples = 10).

Item	KAUH		Princess Basma Hospital		Yarmouk Hospital		Total			
	Yes	No	Yes	No	Yes	No	Yes	%	No	%
Are doors lead-shielded?	√		√		√		10	100%	0	0%
Is there a lead apron?	√		√		√		10	100%	0	0%
Is there an air conditioner?	√		√		√		10	100%	0	0%
Is there adequate light?	√		√		√		10	100%	0	0%
Are there lead protective glasses?	√		√		√		10	100%	0	0%
Are there radiation warning signs on the door?	√		√		√		10	100%	0	0%
Is there gonadal shielding?	√		√		√		10	100%	0	0%
Is there ventilation in the room?	√		√		√		10	100%	0	0%
Are room walls shielded with lead-thickness ≥ 3 mm?	√		√		√		10	100%	0	0%
Is the room checked by competent authorities?	√		√		√		10	100%	0	0%
Is the height of lead walls ≥ 2 m?	√		√		√		10	100%	0	0%

**Table 8:** Checklist for structure of MRI at public hospitals. (No. of samples = 10).

Item	KAUH		Princess Basma Hospital		Total			
	Yes	No	Yes	No	Yes	%	No	%
Is there a control console area?	√		√		10	100%	0	0%
Is control console area ≥ 10 m^2^?	√		√		10	100%	0	0%
Is a room size ≥ 42 m^2^?	√		√		10	100%	0	0%
Is there a toilet inside the room?	√		√		10	100%	0	0%
Is there a headphone for patient?	√		√		10	100%	0	0%
Is there a communicating system “intercom/ loudspeaker” between patient and RT?	√		√		10	100%	0	0%
Is there a change cubicle?	√		√		10	100%	0	0%
Is there a special form for procedures?	√		√		10	100%	0	0%
